# Supplementary material for: Prevalence and determinants of unhealthy feeding practices among young children aged 6–23 months in five sub-Saharan African countries
Source: PLoS One. 2025 Jan 15;20(1):e0317494. doi: 10.1371/journal.pone.0317494 (PMC11734912; doi:10.1371/journal.pone.0317494)
Supplement: Attachment — Submitted filename: Point to point response_3.docx [file pone.0317494.s003.docx]

**Response to reviewer’s comments**

**Manuscript id:** PONE-D-24-42057

**Title**: Prevalence and determinants of unhealthy feeding practices among young children aged 6–23 months in five sub-Saharan African countries

**Journal**: PLOS ONE

**Subject**: **Submission of revised manuscript**

**Response to editor**

**Response to reviewer 1**

**Q1.** The authors addressed all the comments; however, the absence of the description for the first mention of the abbreviations SSA and USAID was still observed. I suggest including the full description upon first mention and using only the abbreviations throughout the remainder of the text, which poses no issue.

**Response**: Dear reviewer thank you for your through and deep review of our manuscript. Your concern has been addressed. Kindly refer to the revised manuscript page 3 line numbers 61 & 71 for the first use of USIAD and SSA. Please also refer to the entire manuscript on the consistent use of SSA to describe sub-Saharan Africa. Thank you.

Kind regards

Berhan Tekeba (corresponding author) on behalf of all authors.
